# MICRA: Microstructural image compilation with repeated acquisitions

**DOI:** 10.1016/j.neuroimage.2020.117406

**Published:** 2021-01-15

**Authors:** Kristin Koller, Umesh Rudrapatna, Maxime Chamberland, Erika P. Raven, Greg D. Parker, Chantal M.W. Tax, Mark Drakesmith, Fabrizio Fasano, David Owen, Garin Hughes, Cyril Charron, C John Evans, Derek K. Jones

**Affiliations:** a Cardiff University Brain Research Imaging Centre (CUBRIC), School of Psychology, Cardiff University, Cardiff, CF24 4HQ, United Kingdom; b Mary MacKillop Institute for Health Research, Australian Catholic University, Melbourne, Australia; c Experimental MRI Centre (EMRIC), School of Biosciences, Cardiff University, Cardiff, United Kingdom; d Siemens Healthcare GmbH, Erlangen, Germany

## Abstract

We provide a rich multi-contrast microstructural MRI dataset acquired on an ultra-strong gradient 3T Connectom MRI scanner comprising 5 repeated sets of MRI microstructural contrasts in 6 healthy human participants. The availability of data sets that support comprehensive simultaneous assessment of test-retest reliability of multiple microstructural contrasts (i.e., those derived from advanced diffusion, multi-component relaxometry and quantitative magnetisation transfer MRI) in the same population is extremely limited. This unique dataset is offered to the imaging community as a test-bed resource for conducting specialised analyses that may assist and inform their current and future research. The Microstructural Image Compilation with Repeated Acquisitions (MICRA) dataset includes raw data and computed microstructure maps derived from multi-shell and multi-direction encoded diffusion, multi-component relaxometry and quantitative magnetisation transfer acquisition protocols. Our data demonstrate high reproducibility of several microstructural MRI measures across scan sessions as shown by intra-class correlation coefficients and coefficients of variation. To illustrate a potential use of the MICRA dataset, we computed sample sizes required to provide sufficient statistical power *a priori* across different white matter pathways and microstructure measures for different statistical comparisons. We also demonstrate whole brain white matter voxel-wise repeatability in several microstructural maps. The MICRA dataset will be of benefit to researchers wishing to conduct similar reliability tests, power estimations or to evaluate the robustness of their own analysis pipelines.

## 1 Introduction

The primary aim of this work was to collect, and disseminate to the neuroimaging community, the MICRA (Microstructural Image Compilation with Repeated Acquisitions) data set - a unique and rich multivariate (diffusion, relaxometry, magnetisation transfer) microstructural MRI archive that allows variance and co-variance of measures to be estimated between tracts, between multiple time-points and between different individuals.

To provide just one example of the utility of such a dataset, we present estimates of sample size calculations that could inform current or planned future microstructural imaging experiments. With the movement towards “open science” practices (Allen and Mehler, 2019; Munafò et al., 2017), there is increasing demand to demonstrate *a priori* that study designs are adequately powered to answer a targeted question. In turn, this requires an assessment of test-retest repeatability as input to the sample size estimations. There is also a trend to complement diffusion-based microstructural measurements with additional measures that have enhanced sensitivity to myelin, including those derived from relaxometry and magnetisation transfer-based approaches (Ercan et al., 2018; Friedrich et al., 2020; Geeraert et al., 2019; Jung et al., 2018; Lipp et al., 2019; Metzler-Baddeley et al., 2017; Morris et al., 2020; Uddin et al., 2019). While it is appealing to collect data across the gamut of *available* options, each new contrast takes time (and invokes real cost) to acquire, and it is important to establish that the measurements are reproducible in themselves, and to establish their reproducibility so that, if necessary, one might prioritise certain image contrasts over others. There is quite an extensive literature on the reproducibility of single neuroimaging measures (e.g., Papinutto et al., 2013; Stikov et al., 2015; Biswal et al., 2010; O'Connor et al., 2017; Vollmar et al., 2010; Grech-Sollars et al., 2015; Prčkovska et al., 2016, Tong et al., 2019), focusing predominantly on inter-site reproducibility and with some focusing on within-site reproducibility. However, to the best of our knowledge, comparison of the reproducibility of multiple microstructural imaging measures, measured in the same population, does not exist at the time scale and sampling frequency presented here.

The resource provided here to the community addresses this gap, allowing for detailed assessment of the reliability of measures derived from optimised multi-shell diffusion, multi-component relaxometry and quantitative magnetisation transfer acquisition protocols. Here we compute reliability statistics (intra-class correlation and coefficient of variation) across three example tracts and individual measures from each microstructural imaging approach. Although these are illustrative examples, these data could be reprocessed and used to compute other parameters from diffusion, relaxometry and quantitative magnetisation transfer (QMT) models. We provide protocols that can be used for power calculations highlighting the utility of the resource to researchers wishing to conduct similar reliability tests/ power calculations. However, this rich, high quality data resource, acquired on an ultra-strong-gradient Connectom 3T system that may not otherwise be readily accessible, will also be of value to those developing and evaluating new data-processing approaches (e.g., denoising, clustering, segmentation, joint-estimation and tractography algorithms).

## 2 Method

### 2.1 Participants

Six neurologically healthy adults (age range 24–30, 3 males and 3 females) were recruited from Cardiff University's staff and student panels. Screening for safety eligibility to undergo MRI scanning was conducted and participants received monetary compensation for participation. All participation was contingent upon prior written informed consent and ethical approval for this study was granted by Cardiff University's School of Psychology ethics committee.

### 2.2 MRI hardware: ultra-strong gradient 3T

Whole brain MRI data were acquired using an ultra-strong gradient (300mT/m) 3T Connectom research only MRI scanner, a modified 3T MAGNETOM Skyra (Siemens Healthcare, Erlangen, Germany). Compared to standard MRI gradients (45–80mT/m), the Connectom gradients allow for shorter diffusion times for a given diffusion weighting resulting in shorter minimum TEs (greater signal to noise ratio) and increased sensitivity to small water displacements (Jones et al., 2018; Setsompop et al., 2013).

### 2.3 MRI data acquisition

Each MRI session lasted approximately 45 min (CHARMED = 18 min, QMT = 12 min, McDESPOT = 11 min) and was repeated 5 times within a two-week period. Care was taken to avoid potential diurnal effects by performing scans for each participant at approximately the same time of day (i.e., within 1–2 h of the same scan start time-of-day).

The MRI protocol included the following sequences:

(i) Multi-shell diffusion-weighted MRI: single-shot spin echo, echo planar imaging data were acquired with both anterior-posterior (AP) and posterior-anterior (PA) phase-encoded directions. The AP-encoded data comprised of two shells of 20 directions (uniformly-distributed according to Jones et al., 1999) at *b* = 200 s/mm^2^ and 500 s/mm^2^, one shell of 30 directions at *b* = 1200 s/mm^2^ and three shells of 61 directions at each of *b* = 2400s/mm^2^, 4000 s/mm^2^ and 6000 s/mm^2^, with two leading non-diffusion-weighted images and a further 11 non-diffusion-weighted images, starting at the 33rd volume, and repeating every 20th volume thereafter. In the PA-encoded data, two non-diffusion-weighted images were acquired. The field of view was 220 × 220 mm in plane, the matrix size was 110 × 110 × 66, reconstructed to a 110 × 110 × 66 image resulting in 2 × 2 × 2 mm^3^ isotropic voxels. The TR and TE were 3000 ms and 59 ms, respectively (for all b-values), and the diffusion gradient duration and separation were 7 ms and 24 ms, respectively.
(ii) Multi-component relaxometry: data were acquired thanks to prototype sequences implementing the McDESPOT protocol (Deoni et al., 2008) with the following parameters: FOV: 220 × 220 × 178.88, matrix size: 128 × 128 × 104 and 1.72 × 1.72 × 1.72 mm^3^ isotropic voxels, SPGR: TR: 4 ms, TE: 1.9 ms, 8 flip angles (3, 4, 5, 6, 7, 9, 13 and 18°), SPGR-IR: TR: 4 ms, TE: 1.9 ms, TI: 450 ms, Flip angle: 5°, full k-space acquisition in PE and slice directions, SSFP: TR: 4.6 ms, TE: 2.3 ms, Flip angles (10, 13.33, 16.67, 20, 23.33, 30, 43.33 and 60°), SSFP180: as SSFP, but with 180° RF phase increments every TR.
(iii) Optimised Quantitative Magnetisation Transfer (QMT, Mougin et al., 2010) data were acquired by using a prototype turbo-flash sequence with parameters: FOV: 220 × 220 × 178.88, matrix size: 128 × 128 × 104, resolution: 1.72 × 1.72 × 1.72 mm^3^ isotropic voxels isotropic, turbo factor 4, radial reordering, non-selective excitation MT pulse duration: 15.36 ms, 11 MT-weighted volumes and 1 vol. without MT-weighting, 11 Frequency offsets (Hz) and 11 flip angles (degrees): 47,180 (628); 56,360 (332); 12,060, (628); 1000 (332);1000 (333); 2750 (628); 2770 (628); 2790 (628); 2890 (628); 1000 (628); 1000 (628) (Cercignani and Alexander, 2006).

### 2.4 MRI data pre-processing and processing

Multi-shell diffusion-weighted data were pre-processed using a custom in-house pipeline comprising tools from both the FSL (Andersson et al., 2003; Andersson and Sotiropoulos, 2016) and MRTrix (Veraart et al., 2016; Vos et al., 2017) software packages and in-house software. Specifically, AP- and PA-encoded images were separately denoised (MRTrix dwidenoise, Veraart et al., 2016) and drift corrected (Vos et al., 2017), then merged (with incorporated EPI, susceptibility and motion correction; FSL *topup* (Andersson et al., 2003) and eddy (Andersson et al., 2016)) corrected for gradient non-linearity distortions (Glasser et al., 2013) with spatio-temporal b-value/vector tracking (Rudrapatna et al., 2018), and finally corrected for Gibbs ringing artefacts (MRTrix mrdegibbs, Kellner et al., 2016). Subsequent processing involved computation of: (i) free-water corrected fractional anisotropy (FA), mean diffusivity (MD) and radial diffusivity (RD) maps (Hoy et al., 2014) from diffusion tensor MRI using the *b* = 1000 s/mm^2^ shell (linear least squares estimation with outlier rejection, Chang et al., 2005). Fibre Orientation Distribution Functions (fODFs) were derived from multi-shell multi-tissue Constrained Spherical Deconvolution (MSMT-CSD, Jeurissen et al., 2014). Microstructural parameters were estimated from all diffusion shells using the CHARMED model (Assaf et al., 2004) using a nonlinear regression routine employing the Levenberg–Marquardt optimization algorithm.

For McDESPOT data, motion correction was applied to the SPGR and SSFP data using FSL mcFLIRT (Jenkinson et al., 2002) followed by brain extraction (Smith, 2002). The QUIT toolbox (Wood, 2018) was utilised for all subsequent fitting. The DESPOT2-FM model was fitted to estimate a B0 map (Deoni, 2009), which was used as input for a final fitting to the 3-pool mcDESPOT model (Deoni et al., 2013), modelling myelin, extra-cellular and CSF contributions using the ‘qimcdespot’ function in QUIT.

QMT data were processed using the QUIT (Wood, 2018) toolbox using the Ramani model (Ramani et al., 2002). For QMT, the MT-weighted volumes were aligned to the non-MT contrast for motion correction and bias correction with B1 maps were applied by computing the B1 field correction based on the field estimate from the fifth MT volume, which was subsequently applied to all MT volumes (FSL FAST, Zhang et al., 2001).

### 2.5 White matter microstructure measures

**Fig. 1:**
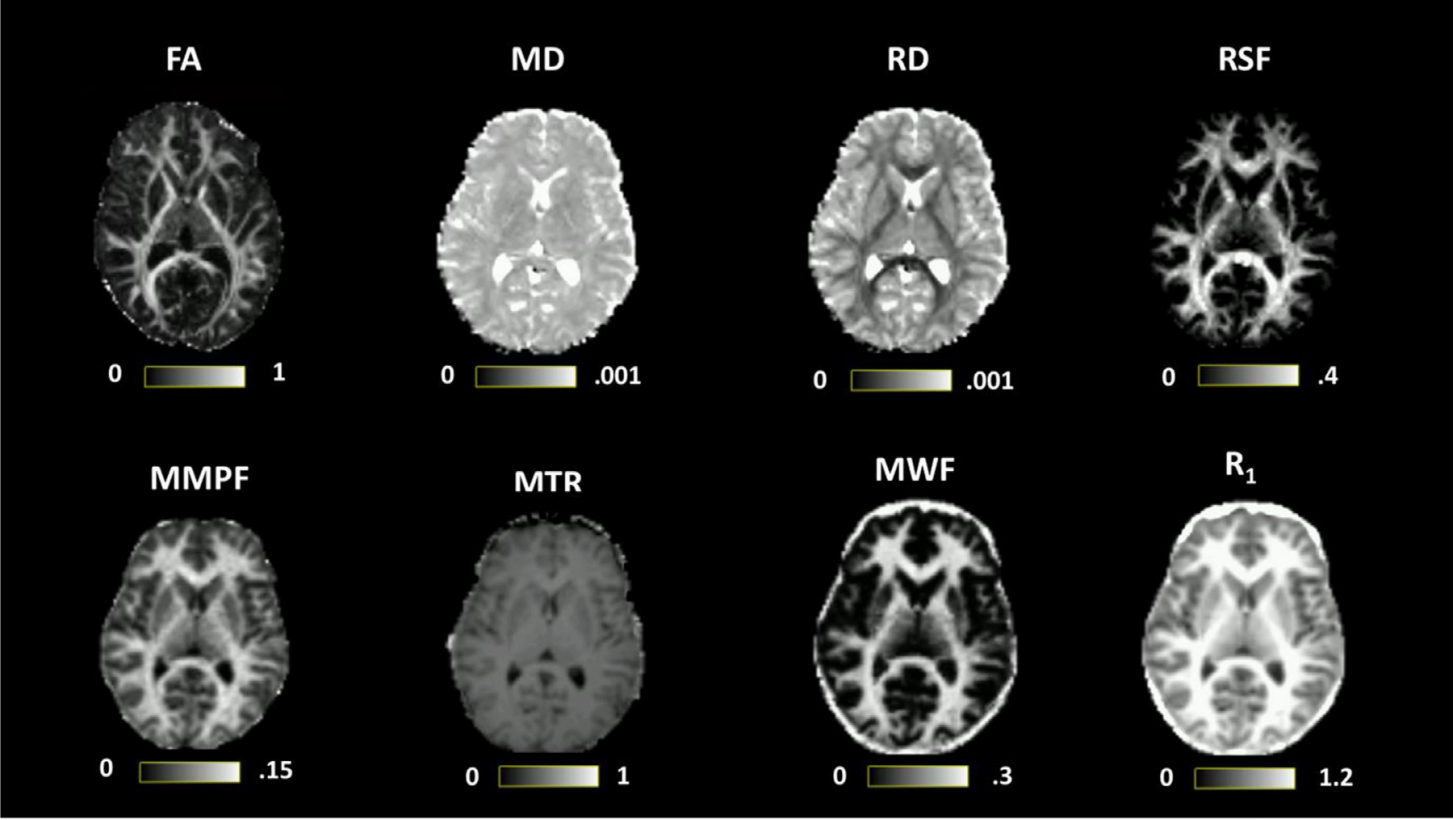
Microstructural maps including FA (fractional anisotropy), MD (mean diffusivity, units = mm^2^/s), RD (radial diffusivity, units = mm^2^/s), RSF (restricted diffusion signal fraction), R_1_ (longitudinal relaxation rate, units = s^−1^), MWF (myelin water fraction), MMPF (macromolecular proton fraction) and MTR (magnetisation transfer ratio) computed for one representative participant. All contrasts are registered to diffusion space.

The following microstructural measures were computed in each voxel: restricted diffusion signal fraction (RSF) fitted from CHARMED (nonlinear regression routine employing the Levenberg–Marquardt optimization algorithm, Assaf et al., 2004), fractional anisotropy (FA), mean diffusivity (MD) and radial diffusivity (RD) from diffusion tensor MRI; myelin water fraction (MWF) and longitudinal relaxation rate (R_1_) from the McDESPOT pipeline (Deoni and Kolind, 2015), the macromolecular proton fraction (MMPF) fitted from the QMT pipeline (Wood, 2018; Ramani et al., 2002) and magnetisation transfer ratio (MTR) computed using home-grown code. Quantitative maps were subsequently linearly registered to the space in which the diffusion MRI data were acquired (‘native space’) using FSL FLIRT (see Fig. 1 for illustration of all maps). Selections of models here are illustrative. The magnitude-reconstructed raw data are also included in the MICRA dataset, enabling researchers to explore other modelling options.

### 2.6 Virtual dissection of tracts

To assess test-retest repeatability, a white matter projection tract (cortico-spinal), association tract (arcuate fasciculus) and the fornix were virtually dissected from whole brain white matter maps for each participant at each time point with probabilistic tractography (MRTrix iFOD2, 1000 seeds x 5000 streamlines, step size = 0.5 × 2 mm^3^ voxel size, angular threshold = 90° x step size/voxel size, fODF threshold = 0.05, Jeurissen et al., 2014, Fig. 2). The fornix was virtually dissected by placing region of interest masks in the anterior hippocampus and fornix body. The CST and the arcuate fasciculus were dissected using TractSeg (Wasserthal et al., 2018) using code available at https://github.com/MIC-DKFZ/TractSeg/. Tractography was conducted in each individual subject's diffusion space. Track density maps (TDI, Calamante et al., 2010) of the resultant tracts were computed and thresholded to exclude voxels through which streamlines passed less than 20 percent. As an *a priori* choice, the analysis was restricted to three tracts in order to show a demonstration of repeatability in one association, one projection and one commissural pathway.

**Fig. 2:**
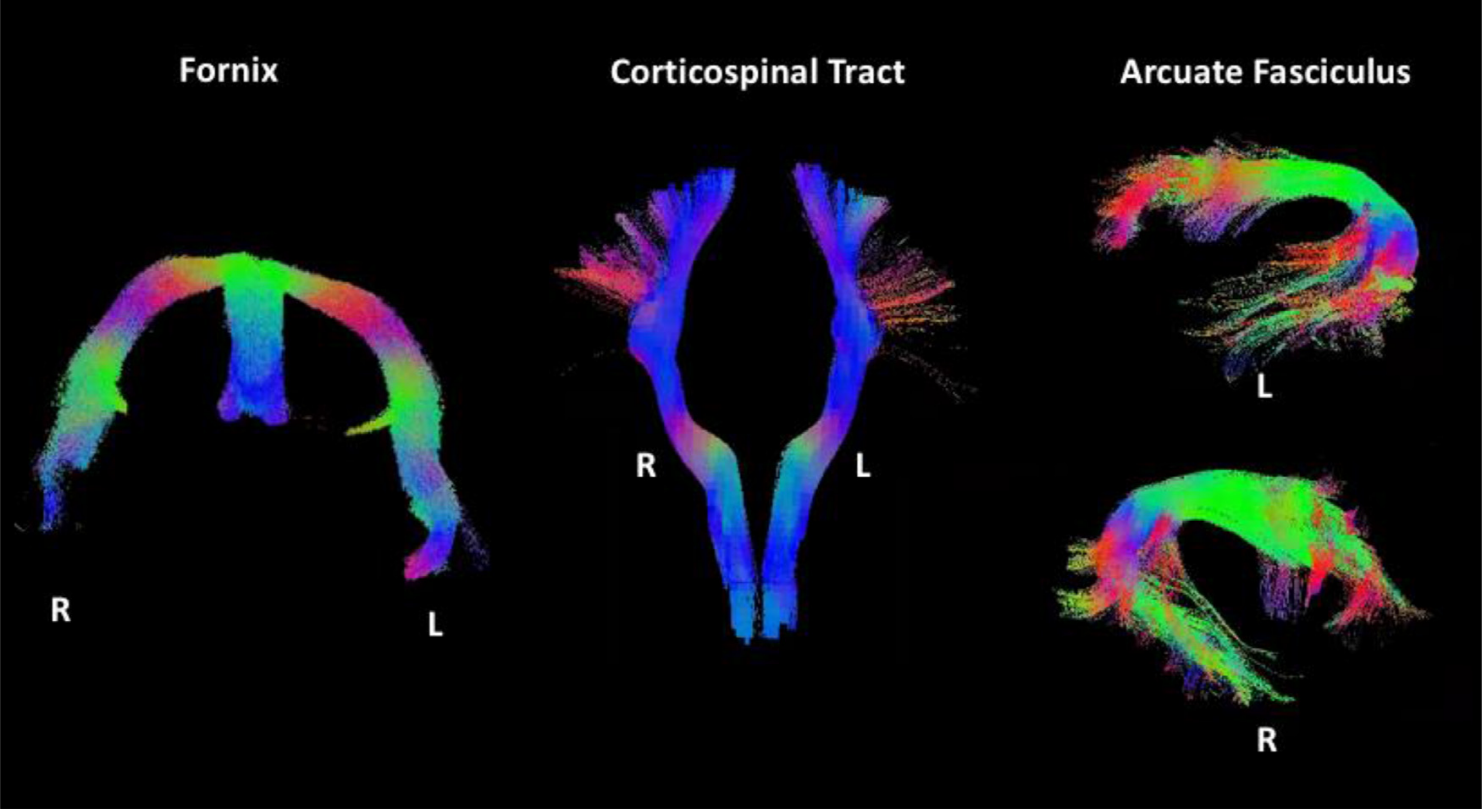
Virtual dissections of the fornix, cortico-spinal tract and arcuate fasciculus dissections in one representative participant (see Methods).

### 2.7 Repeatability at the tract-level

Measures were extracted for each vertex in each streamline and averaged along each tract for statistical comparison. The intra-class correlation coefficient (two-way mixed, absolute agreement) and coefficient of variation were computed for average assessment of test-retest repeatability (Table 1) across all repeated scans.

**Table 1:** Test-retest reliability statistics for microstructure measures. Units (mean and SD) for MD and RD = 10^−3^ mm^2^/s; units for R_1_ = s^−1^, CV = averaged within subject, ICC = averaged across 5 repeated measures.

Metric	Tract	Mean	SD	CV (%)	ICC	ICC pval	*r*
FA		0.47	0.005	1.0	0.93	<0.0001	0.82
MD		1.00	0.010	0.9	0.94	<0.0001	0.87
RD		1.00	0.004	0.6	0.96	<0.0001	0.88
RSF		0.13	0.003	2.1	0.88	<0.0001	0.65
MMPF	Fornix	0.05	0.001	2.6	0.95	<0.0001	0.85
MWF		0.07	0.003	4.2	0.95	<0.0001	0.87
R_1_		0.77	0.005	0.7	0.97	<0.0001	0.90
MTR		0.35	0.0004	0.1	0.94	<0.0001	0.77
FA		0.55	0.003	0.6	0.97	<0.0001	0.89
MD		0.41	0.002	0.5	0.95	<0.0001	0.81
RD		0.63	0.001	0.2	0.95	<0.0001	0.78
RSF		0.27	0.003	1.2	0.86	0.002	0.52
MMPF	CST	0.09	0.001	1.5	0.97	0.0003	0.74
MWF		0.16	0.003	1.7	0.97	<0.0001	0.95
R_1_		1.04	0.004	0.4	0.98	<0.0001	0.91
MTR		0.41	0.002	0.5	0.78	0.01	0.37
FA		0.47	0.005	1.0	0.95	<0.0001	0.86
MD		0.47	0.005	1.0	0.96	<0.0001	0.85
RD		0.64	0.003	0.5	0.96	<0.0001	0.81
RSF	Arcuate Fasciculus	0.26	0.004	1.7	0.82	0.005	0.72
MMPF		0.10	0.002	2.1	0.92	<0.0001	0.79
MWF		0.16	0.002	1.4	0.98	<0.0001	0.93
R_1_		1.07	0.001	0.1	0.97	<0.0001	0.90
MTR		0.42	0.003	0.6	0.97	<0.0001	0.92

One-way ANOVAs demonstrated no significant differences between ICC computed for individual time point pairs or between average ICC and individual time point ICC comparisons for the CST (F(9,70) = 0.74, *p* = .67) and the Fornix (F(9,70) = 0.19, *p* = .07). Although initially a difference was suggested for the Arcuate Fasciculus (F(9,70) = 2.2, *p* = .03), no comparisons survived correction for multiple comparisons as shown with Tukey post-hoc correction.

Moreover, to ascertain whether there was an effect of time on reproducibility (i.e., do those measurements that are more closely-spaced in time agree better than those spaced further apart in time?), intra-class correlation coefficients were also computed for individual time point pairs across all scan sessions.

### 2.8 Repeatability at the voxel level

While our strong preference for microstructural comparisons is to use a ‘tractometry’-based approach, (Bells et al., 2011; Chamberland et al., 2019) to perform individual/ group microstructural comparisons, we recognise the prevalence of voxel-based analyses. We therefore conducted a separate analysis of the reproducibility of each metric at the voxel-level within white matter across the whole brain. This was done by adopting the white matter skeletonisation approach popularised in the TBSS (Tract-Based Spatial Statistics, Smith et al., 2006, framework, part of FSL (Smith et al., 2004). First, and as above, the MMPF, R_1_, MTR and MWF maps for a given participant and timepoint were first registered to the individual's native diffusion space using FLIRT (Jenkinson and Smith, 2001).

Then, FA maps from all subjects at all time-points were aligned into a common space using the nonlinear registration tool FNIRT (Andersson et al., 2007; Andersson et al., 2007) which uses a b-spline representation of the registration warp field (Rueckert, 1999). Next, the mean FA image was created and thinned to create a mean FA skeleton which represents the centres of all tracts common to the group. Each subject's aligned FA map was then projected onto this skeleton in MNI space. The nonlinear warps and skeleton projections generated for FA were applied to the corresponding non-FA maps (already in diffusion space) to create white matter skeletons in MNI space for these additional metrics. Prior to analysis, a further thresholding step was applied. Specifically, each voxel in the skeletonised data was only retained for further analysis if, in that voxel, all 5 participants at all six time-points had an FA > 0.2. This was to provide enhanced assurance that the analysis was restricted to white matter. For each metric, the Pearson correlation was then conducted across all voxels in the thresholded skeleton between each possible pair of time-points to assess the repeatability across whole brain white matter.

**Fig. 4:**
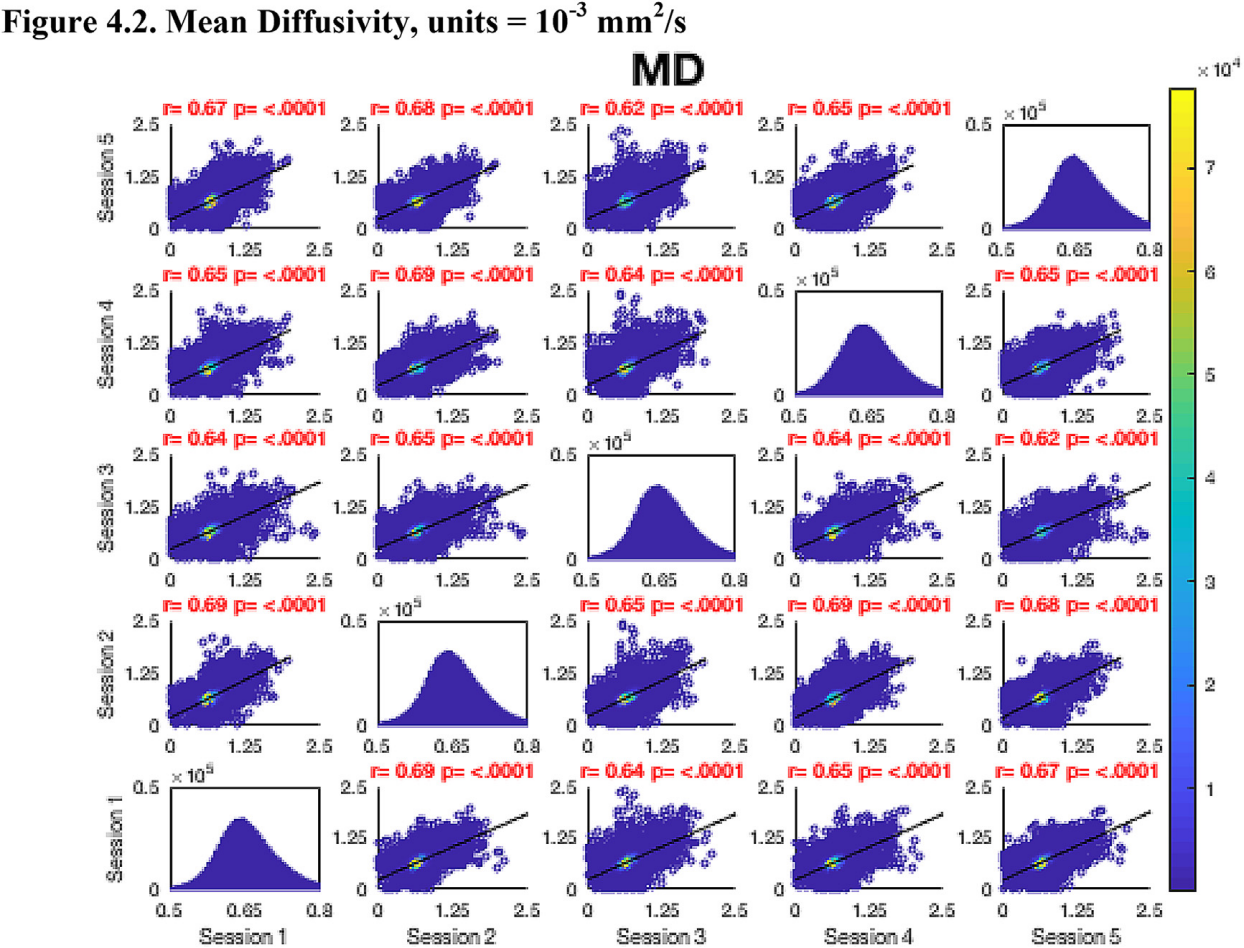
Continued.

**Fig. 4:**
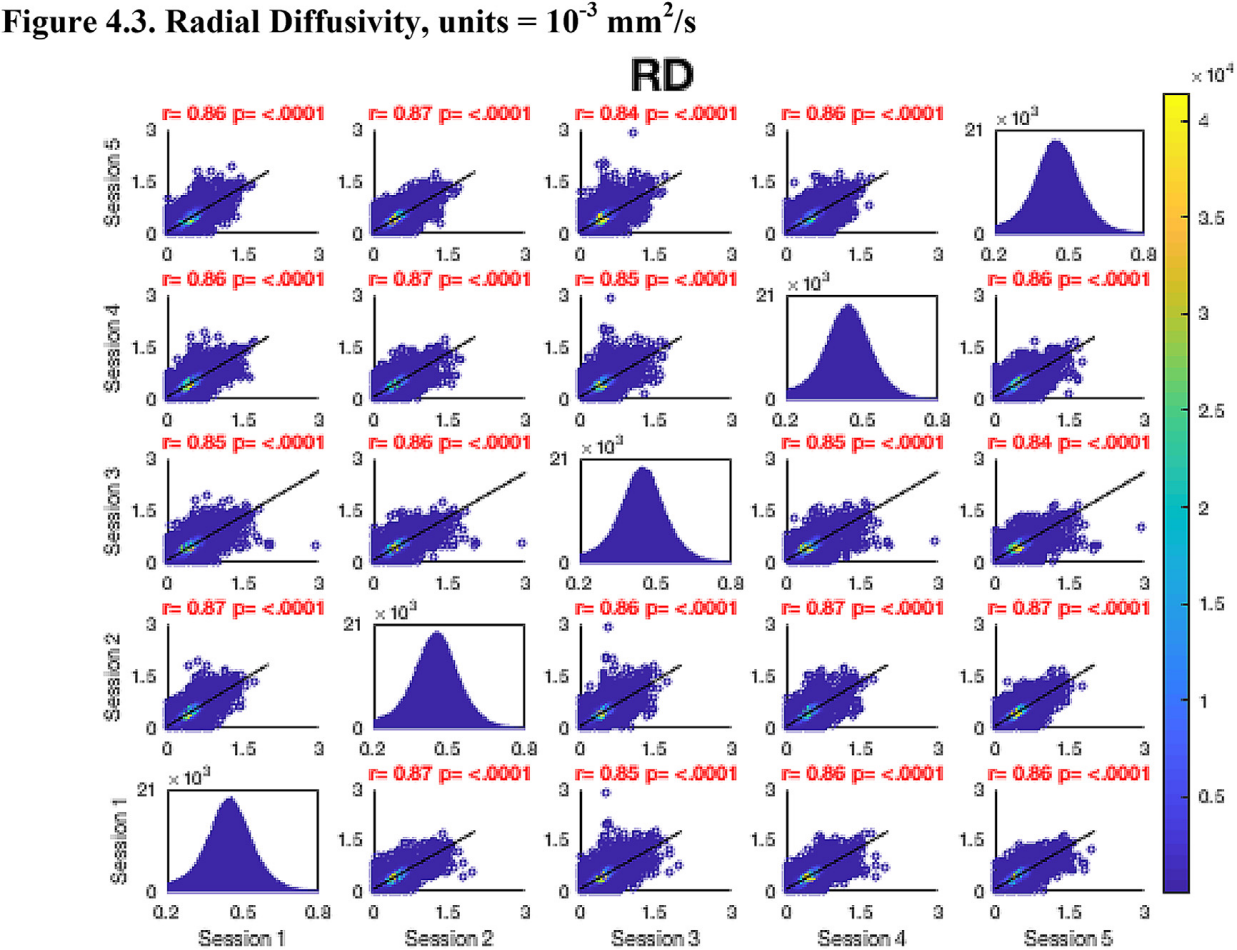
Continued.

**Fig. 4:**
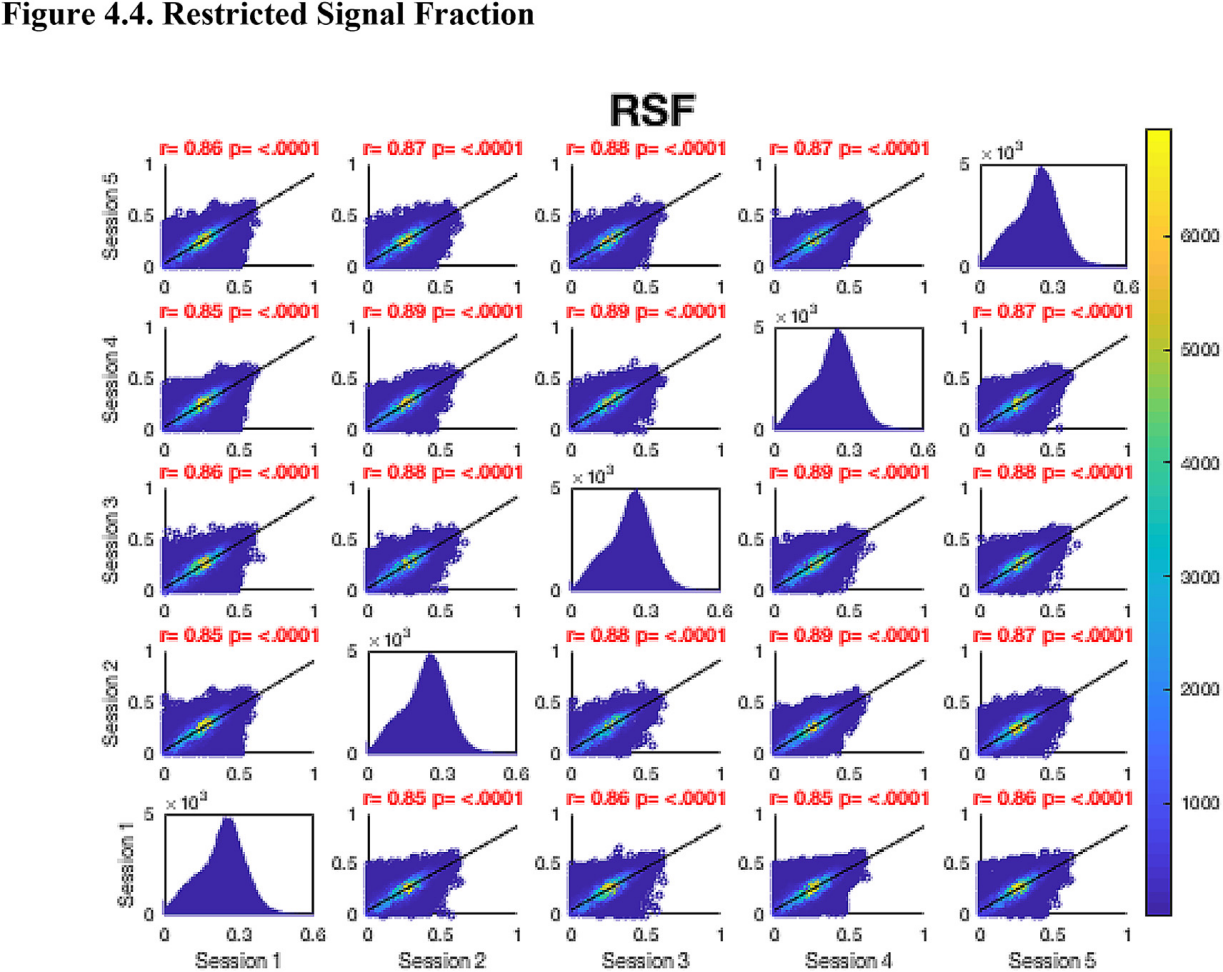
Continued.

**Fig. 4:**
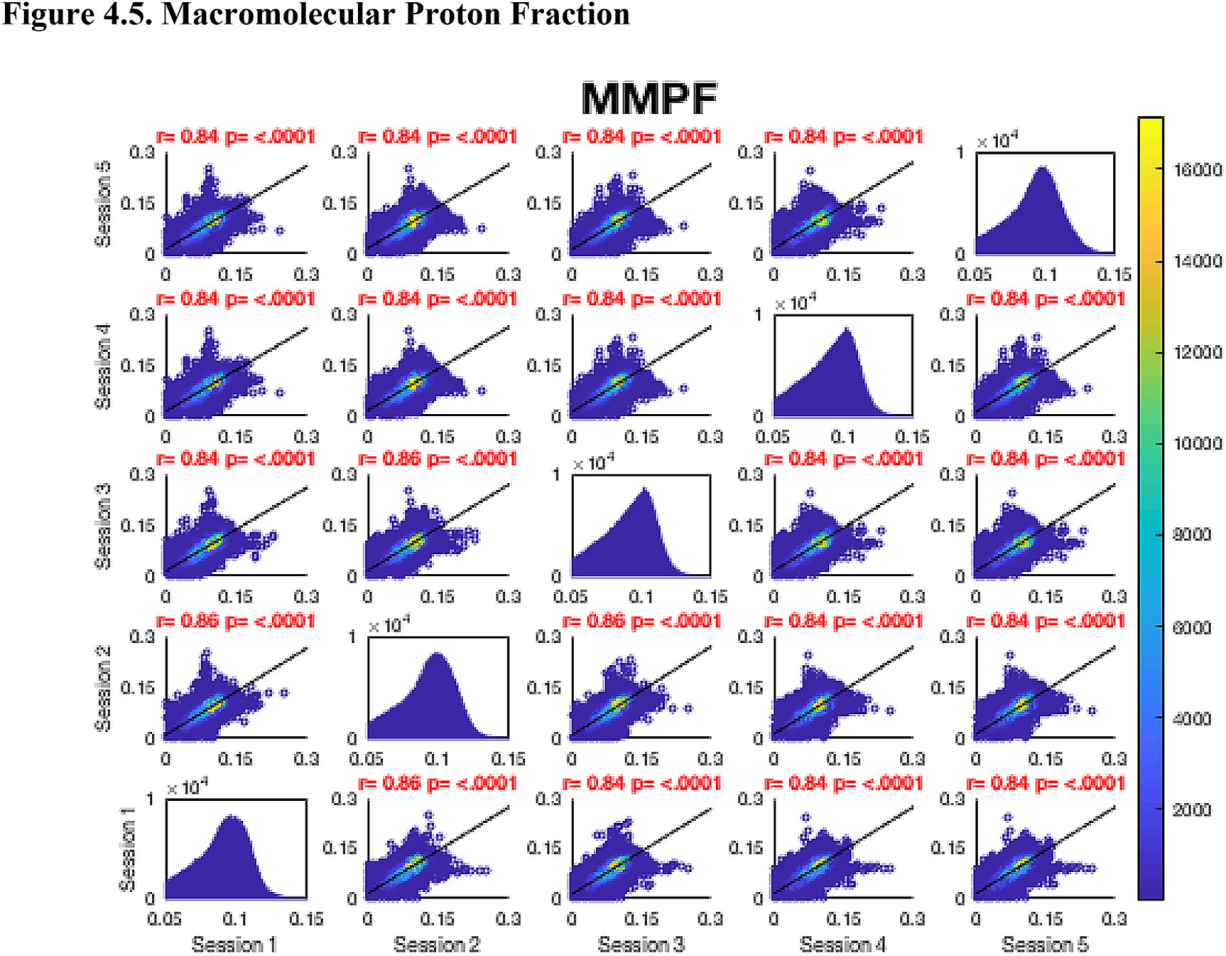
Continued.

**Fig. 4:**
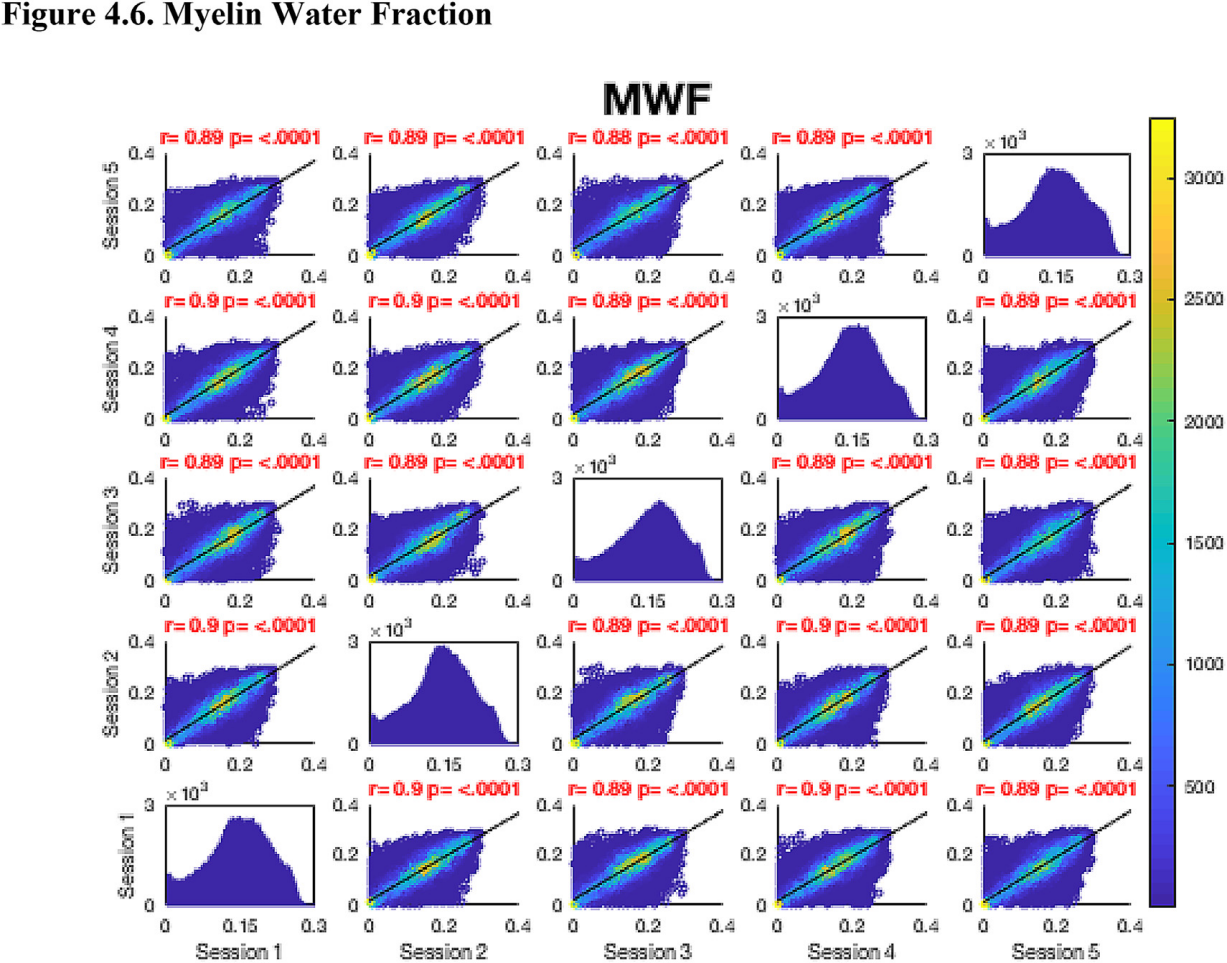
Continued.

**Fig. 4:**
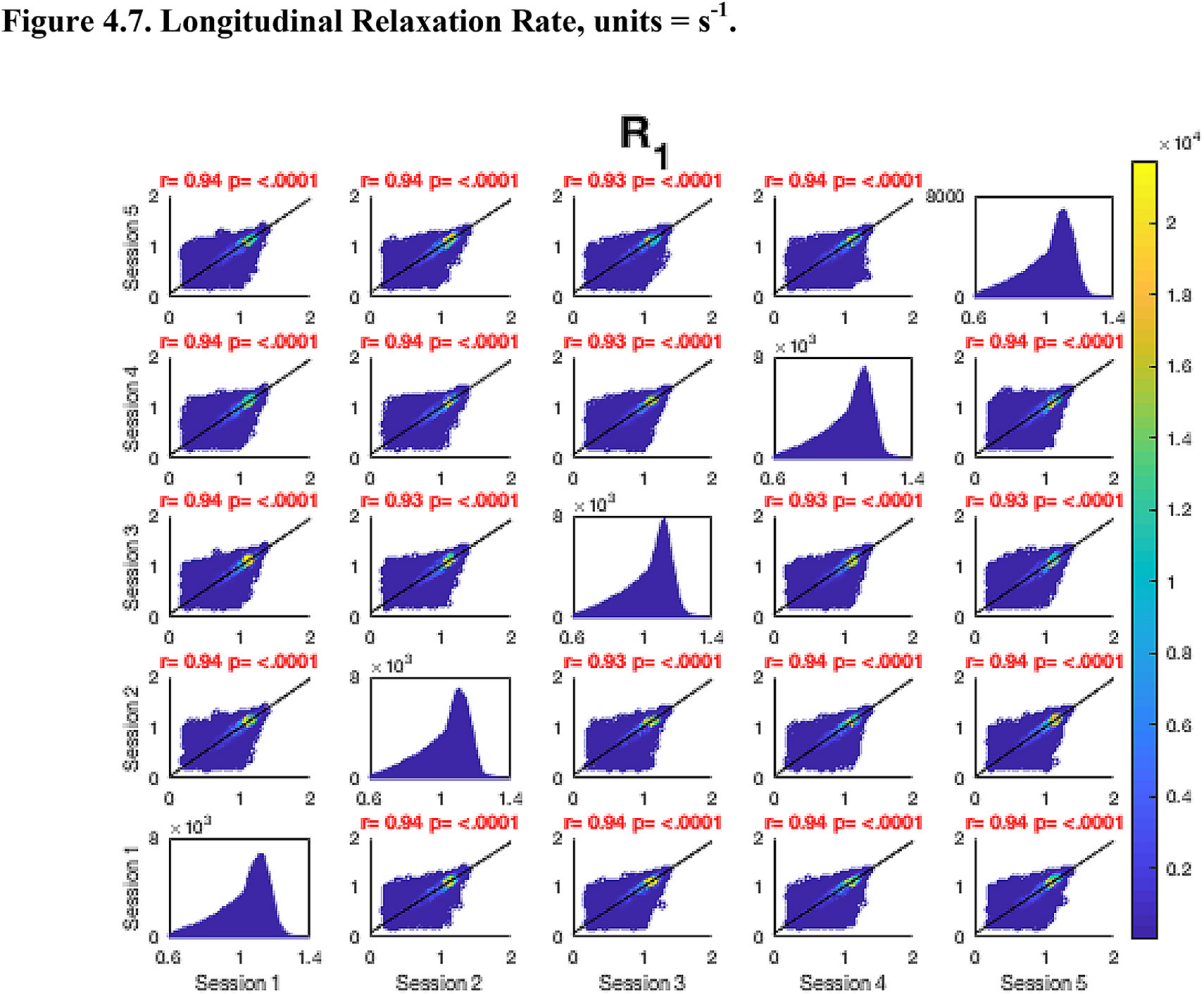
Continued.

**Fig. 4:**
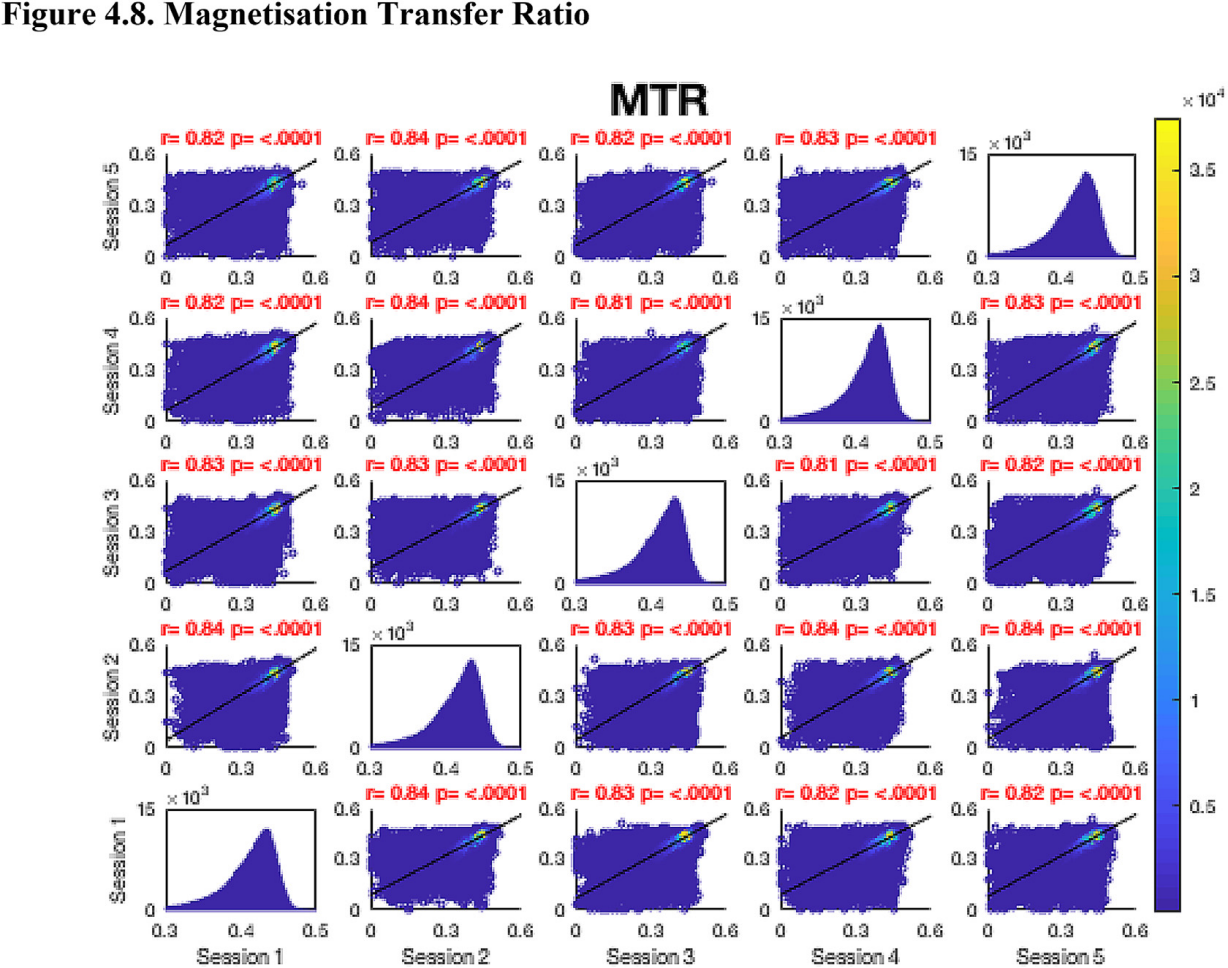
Continued.

**Fig. 5:**
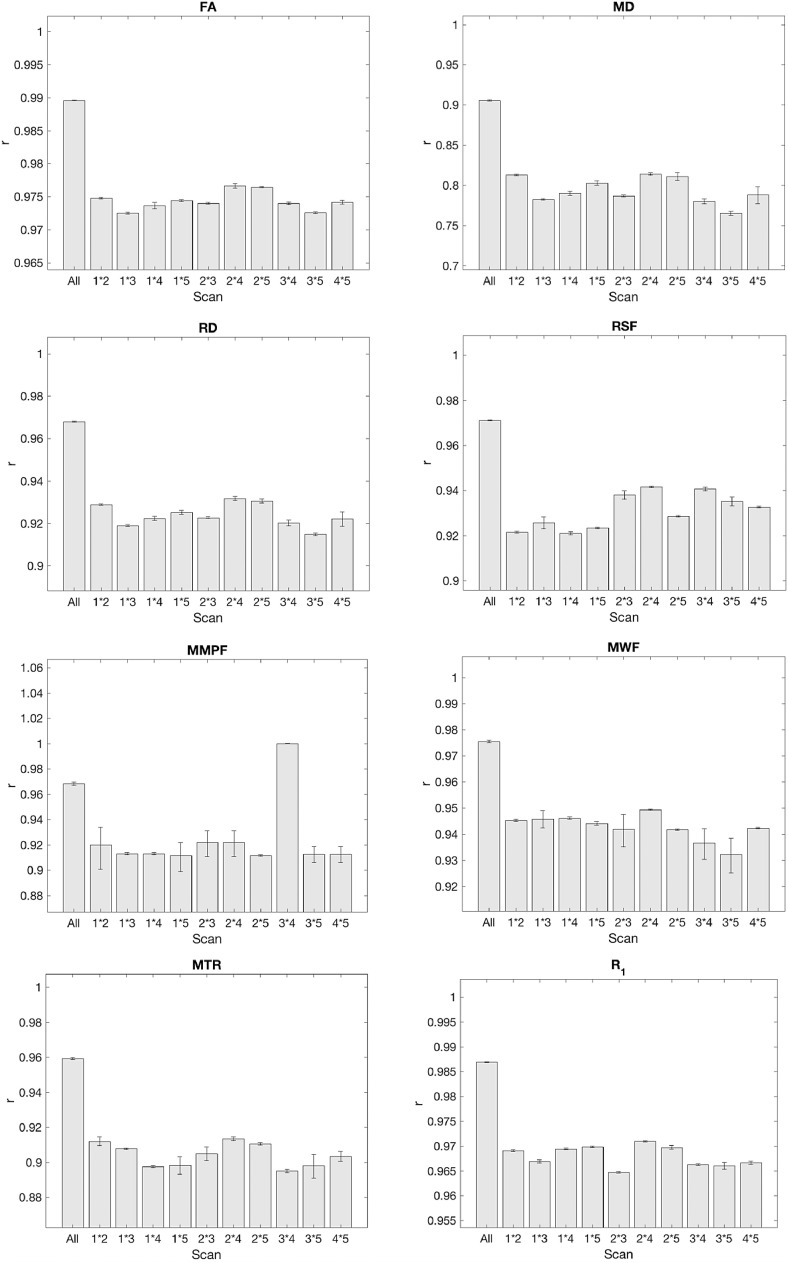
ICC coefficients for whole brain white matter voxels pooled across all participants. Error bars represent the lower and upper bound confidence intervals.

### 2.9 Demonstration of sample size estimation

To further illustrate the utility of the reproducibility data, we consider the minimum number of subjects needed to reach statistical power of 0.8 and significance of *α* = 0.05 for two different types of statistical tests routinely carried out in the neuroimaging literature, as outlined below (power calculations were computed using G*Power, see Supplementary 2, Faul et al., 2009):

(i) *Independent groups t-test (e.g., for comparing a group of patients to a group of healthy controls).* Here, we evaluate the numbers of subjects needed to detect a 1% and 5% group difference in each microstructural metric. This was done for all measures and tracts according to means and standard deviations presented in Table 1 (Fig. 4). The minimum n was computed by inputting the percentage change from the mean and standard deviations into G*Power.
(ii) *Group (2)* *x* *Time (2) between-within groups ANOVA (e.g., for showing that there is a difference in the longitudinal evolution of a metric between two groups).* This was estimated across all measures and tracts at small, medium and large effect sizes (Fig. 5). Pearson correlation coefficients were used to account for the correlation amongst repeated measures for sample size estimation (Table 1).

## 3 Results

Microstructural maps computed for one representative participant are presented in Fig. 1. Fig. 2 shows a typical reconstruction of the fornix, arcuate fasciculus and cortico-spinal tracts, which were successfully dissected bilaterally for each MRI session for each participant. For one participant, calculation of a robust estimate of MWF failed for one session, while for another participant, calculation of the MMPF was not robust in one session. Thus, these values were not included in the analyses.

### 3.1 Repeatability at the tract level

**Fig. 3:**
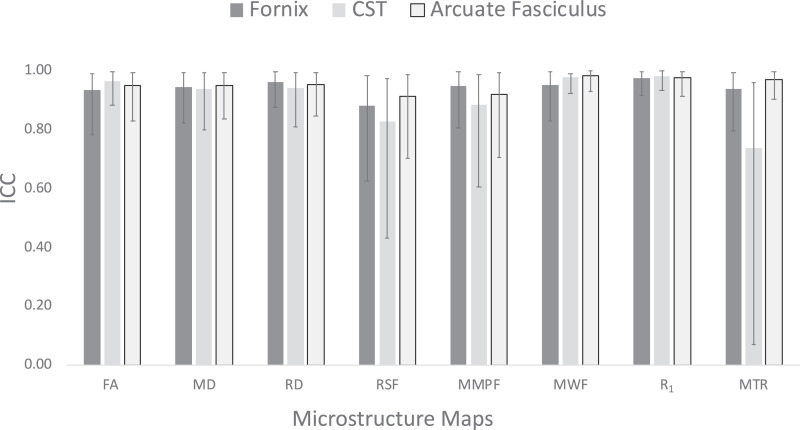
Intra-class correlation coefficients (two-way mixed, absolute agreement) for test-retest repeatability of microstructure measures measured 5 times in 6 participants. ICC = intra-class correlation, CST = cortico-spinal tract, FA = fractional anisotropy, MD = mean diffusivity, RD = radial diffusivity, RSF = restricted diffusion signal fraction, MMPF = macromolecular proton fraction, MWF = myelin water fraction, R_1_ = longitudinal relaxation rate, MTR = magnetisation transfer ratio.

The coefficients of variation (CV) were overall low, (Table 1), ranging from 0.2 to 4.2%. Intra-class correlations ranged from 0.78 to 0.98 with all demonstrating a high degree of repeatability (Table 1, Fig. 3). Estimated sample sizes for an independent groups *t*-test to measure a 1% and 5% group difference are presented in Fig. 6 and for a 2 × 2 between-within ANOVA to measure small, medium and large effect sizes in Fig. 7.

The CV values in Table 1 represent the averaged within-subject coefficients of variation. ICC values presented in Table 1 and Fig. 3 represent the two-way mixed effects, absolute agreement with multiple measurements (Mcgraw and Wong, 1996) estimated by the below equation:

$$\text{ICC}=\frac{MS_{R}-MS_{E}}{MS_{R}+\frac{MS_{C}-MS_{E}}{n}}$$

MS_R_ = mean square for rows; MS_E_ = mean square for error; MS_C_ = mean square for columns; *n* = number of subjects (see Koo and Li, 2016, pp. 157 for detailed breakdown of equations for ICC).

### 3.2 Repeatability at the voxel level

Fig. 4 shows the analysis of repeatability at the voxel-level, for all voxels on the white matter skeleton (see Methods). For each metric, voxelwise whole brain white matter Pearson correlations are presented between individual time points for voxels pooled across all subjects, with a colour scale denoting the number of voxels in each joint histogram bin. Additionally, univariate histograms show the distributions of voxels of a given metric across all voxels.

**Fig. 4:**
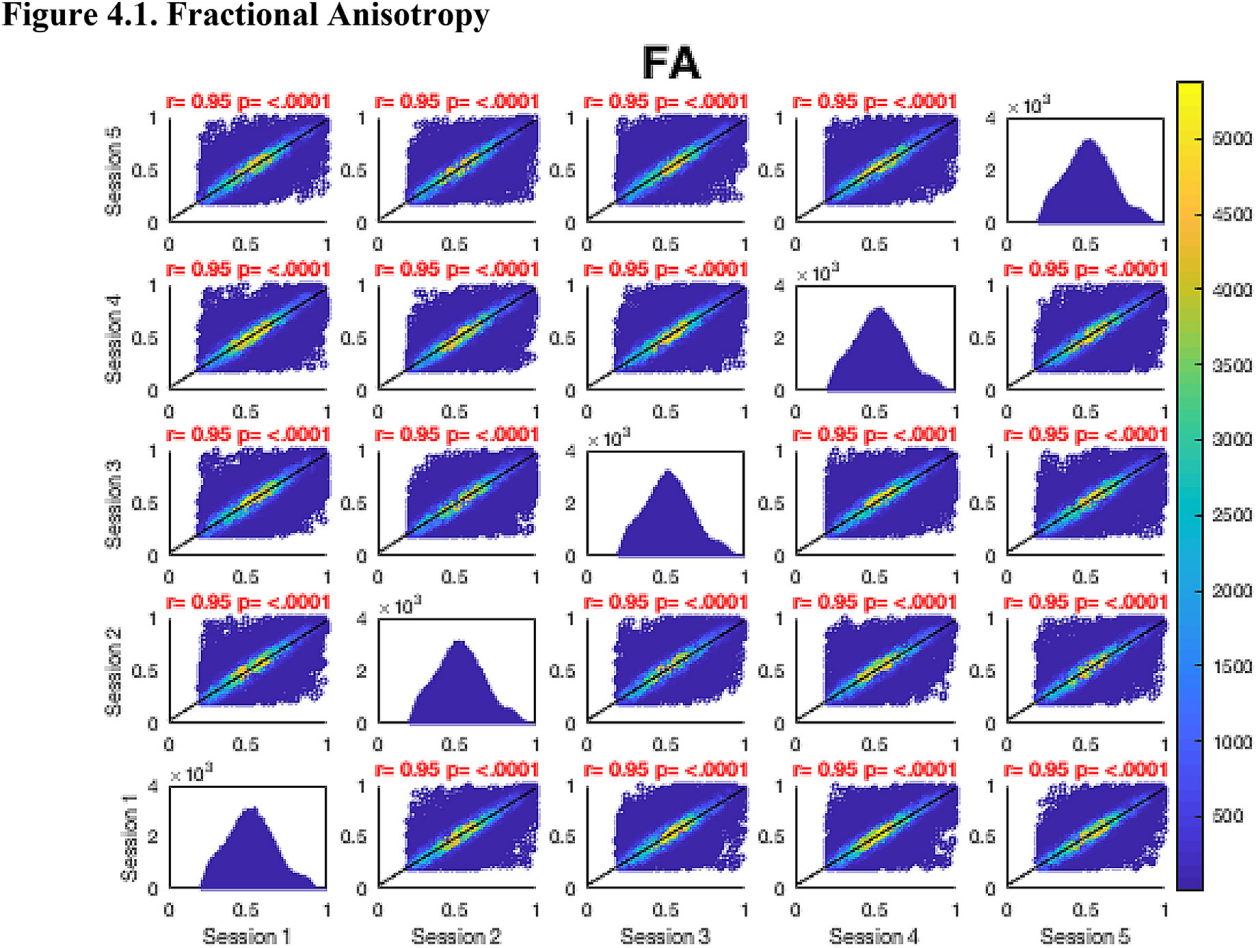
Voxel-wise whole brain white matter Pearson correlations are presented between individual time points for voxels pooled across all subjects, r = Pearson correlation coefficient, p < .0001 for all plots. Fig. 4.1 Fractional Anisotropy (FA), Fig. 4.2 Mean Diffusivity (MD), units = 10^−3^ mm^2^/s, Fig. 4.3 Radial Diffusivity (RD), units = 10^−3^ mm^2^/s, Fig 4.4 Restricted Signal Fraction (RSF), Fig. 4.5 Macromolecular Proton Fraction (MMPF), Fig. 4.6 Myelin Water Fraction (MWF), Fig. 4.7 Longitudinal Relaxation Rate (R_1_), units = s^−1^, Fig. 4.8 Magnetisation Transfer Ratio. For the 2D scatter plots, the colour scale denotes the number of voxels in each joint histogram bin. The univariate histograms  on the diagonal show the distributions of voxels of a given metric across all voxels (subject to the inclusion criteria described in ‘Methods’) for each scan.

In terms of the Pearson correlation coefficient, the most reproducible metric is FA with all pair-wise *r* > 0.95 (which is unsurprising given that the FA was used to drive the skeletonisation process). The heatmap representation of the joint histograms show that, despite some considerable scatter, the vast majority of data points lie along the line of identity. For the other metrics: R_1_ (*r* > 0.93) shows superb reproducibility. RSF (*r*> 0.85), MMPF (*r* > 0.84), MTR (*r* > 0.81), MWF (*r* > 0.88) and RD (*r* > 0.84) also show good performance. The lowest reproducibility is for MD (*r* > 0.62).

To further ascertain whether there was an effect of time on reproducibility (i.e., do those measurements that are more closely-spaced in time agree better than those spaced further apart?), intra-class correlation coefficients were computed for individual time point pairs across all scan sessions (Fig. 5).

### 3.3 Demonstration of sample size estimation

**Fig. 6:**
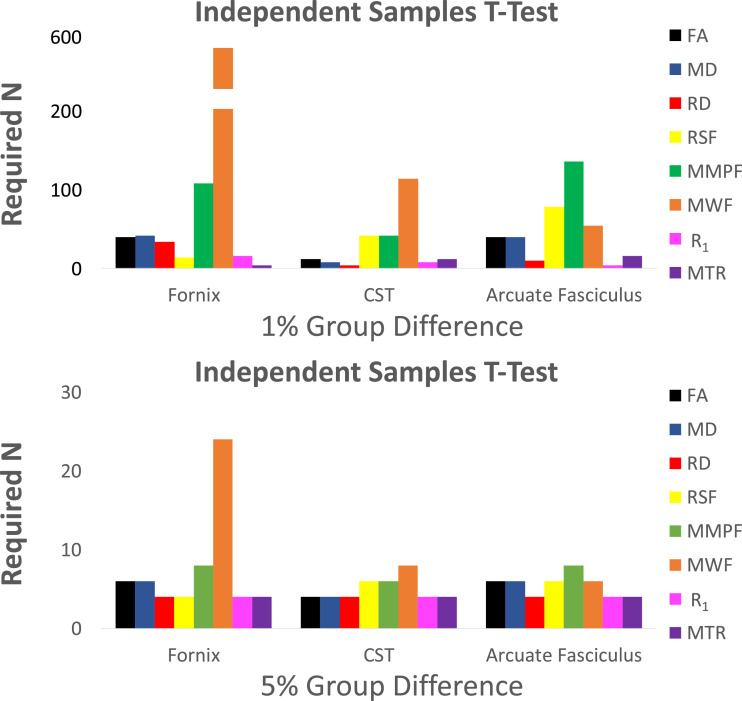
Estimated sample sizes for statistical designs required to reach a power of 0.8 and significance ***α*** of 0.05 for independent groups *t*-test in three white matter tracts across several microstructure measures. Sample sizes were estimated for 1% and 5% group differences according to means and standard deviations presented in Table 1. The standard deviations were assumed to be constant across groups.

**Fig. 7:**
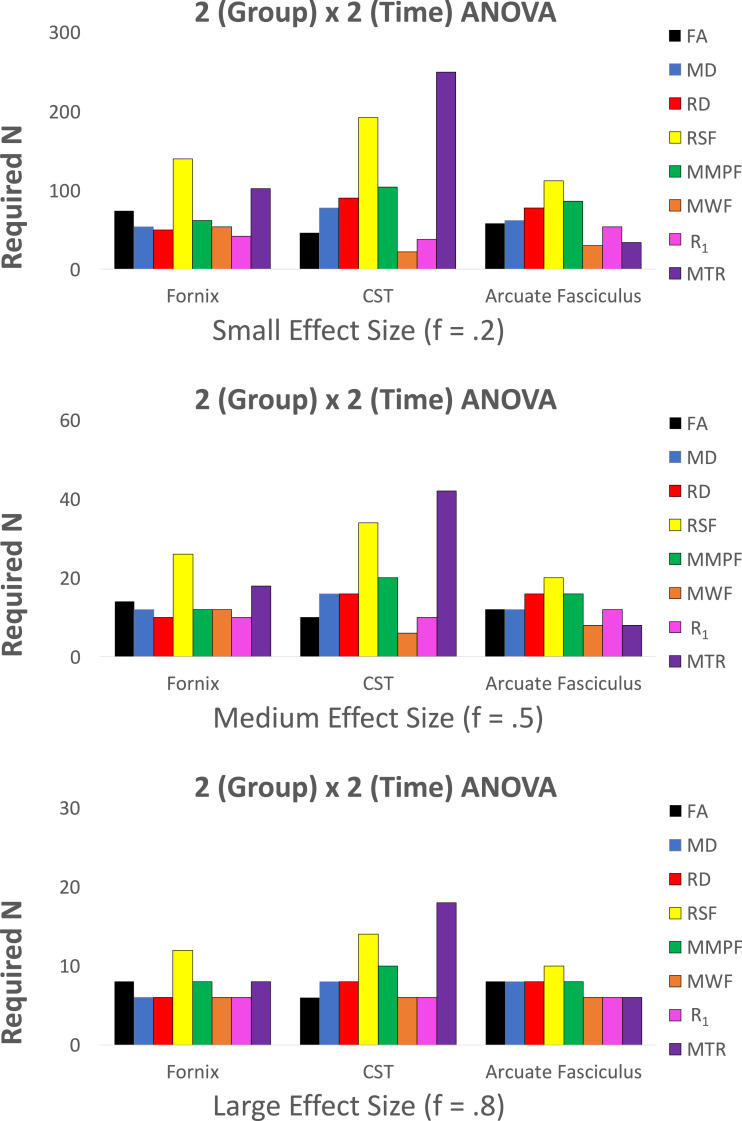
Estimated sample sizes for statistical designs to reach a power of 0.8 and ***α*** of 0.05 in three white matter tracts for a Group (2) x Time Point (2) ANOVA. Pearson correlations between all 5 sessions were averaged by transformation to Fischer's Z (Fisher, 1915) to obtain an average correlation among repeated coefficients for each metric (Table 1). Correlation coefficients were used to estimate required sample sizes for each metric.

Returning to the tract-based estimates, Fig. 6 shows estimated sample sizes for statistical designs required to reach a power of 0.8 and significance *α* of 0.05 for independent groups *t*-test in the fornix, cortico-spinal tract and arcuate fasciculus for the different metrics. Clearly the number of subjects required varies by an order of magnitude depending on which pathway is examined and which metric is used. A similar heterogeneity of required sample sizes is seen when powering for ANOVA analyses (Fig. 7).

## 4 Discussion

We present this paper as an introduction to MICRA – a multi-variate microstructural dataset collected on an ultra-strong gradient Connectom 3T MRI scanner. We offer the MRI community access to this MRI archive as a test-bed for conducting specialised analyses where access to repeated measures of multi-contrast MRI data may help to inform current and future research. As a demonstration of a possible application of our MRI archive, we explored the reproducibility of microstructural measures, including intra-class correlations and coefficients of variation, across multiple white matter tracts. Additionally, we presented estimates of samples sizes required for an independent groups *t*-test and a Group(2) x Time(2) ANOVA to reach statistical power of 0.8 and significance of *α* = 0.05 for various effect sizes across white matter measures and tracts.

Virtual dissections performed with probabilistic tractography (iFOD2, Jeurissen et al., 2014, MRTrix) demonstrated the fornix, the arcuate fasciculus and cortico-spinal tracts in all six participants and were replicated for all of the five repeated MRI scans. The overall low coefficients of variation within participants and the high correlations among repeated measures suggest a high degree of consistency of microstructure measures across repeated tracts and scans.

Sample size estimations performed for an independent group comparison (*t*-test) across microstructure measures and tracts demonstrated similar patterns of required sample sizes for 1% and 5% increase changes, with expectedly larger samples required for demonstrations of 1% change. The differences in the standard deviation of measures are reflected in the different sample sizes required. Notably, MWF in the fornix and in the cortico-spinal tract, and MMPF in the Arcuate Fasciculus demonstrated the largest sample sizes required. Conversely, the RSF, MTR, RD and R_1_ in the fornix, the MTR, FA, MD, RD and R_1_ in the cortico-spinal tract, and the RD, MTR and R_1_ in the arcuate fasciculus demonstrated the smallest required sample sizes. Power analyses to estimate sample sizes for a 2 × 2 between-within ANOVA demonstrated that the measures showed a similar pattern for sample size requirement for the fornix and the cortico-spinal tract. In these tracts, measures requiring the smallest sample size were the MWF and R_1_. Diffusion measures (FA, RD, MD) and the MMPF required larger sample sizes, whereas the MTR and the RSF required the largest sample sizes to reach a given effect size. In contrast, the arcuate fasciculus demonstrated a pattern in which the diffusion measure required larger sample sizes compared to R_1_ and MTR, with MWF requiring the smallest sample size.

To conclude, we present a rich multivariate archive of microstructural MRI data acquired on a Connectom 3T MRI scanner. It is important for researchers to take into consideration that the reproducibility statistics reported here are directly applicable only to scans and analyses that follow conditions unique to the present study conducted on a high gradient Connectom MRI scanner. Although this is unique to the present study, the Connectom-acquired diffusion data offers the highest quality diffusion data available, offering researchers an indication of what might be possible in a ‘best case scenario’.

Data from this study demonstrate that microstructure measures derived from multi-shell diffusion, multi-component relaxometry and quantitative magnetisation transfer acquired on an ultra-strong gradient 3T MRI scanner are reliable as demonstrated by low coefficients of variation and high intra-class correlation coefficients across measures and tracts.

## CRediT authorship contribution statement

**Kristin Koller:** Conceptualization, Data curation, Formal analysis, Methodology, Writing - original draft, Writing - review & editing. **Umesh Rudrapatna:** Conceptualization, Data curation, Formal analysis, Methodology, Writing - review & editing. **Maxime Chamberland:** Formal analysis, Methodology, Writing - review & editing. **Erika P. Raven:** Data curation, Formal analysis, Funding acquisition, Methodology, Writing - review & editing. **Greg D. Parker:** Formal analysis, Methodology, Writing - review & editing. **Chantal M.W. Tax:** Data curation, Formal analysis, Funding acquisition, Methodology, Writing - review & editing. **Mark Drakesmith:** Conceptualization, Data curation, Methodology, Writing - review & editing. **Fabrizio Fasano:** Methodology, Writing - review & editing. **David Owen:** Software, Writing - review & editing. **Garin Hughes:** Software, Writing - review & editing. **Cyril Charron:** Software, Writing - review & editing. **C John Evans:** Conceptualization, Data curation, Formal analysis, Methodology, Writing - review & editing. **Derek K. Jones:** Conceptualization, Formal analysis, Funding acquisition, Methodology, Writing - original draft.

## Appendix Supplementary materials

Image, application 1

Image, application 2

Image, application 3
