# Supplementary material for: MICRA: Microstructural image compilation with repeated acquisitions
Source: Neuroimage. 2021 Jan 15;225:117406. doi: 10.1016/j.neuroimage.2020.117406 (PMC7779421; doi:10.1016/j.neuroimage.2020.117406)
Supplement: Supplementary file 1 [file mmc1.pdf]

Supplementary Table 1. Data shared as part of MICRA

Data Type	Raw (brain extracted nifti)	Processed Maps (in native, diffusion and MNI space)
Multi-Shell Diffusion + CHARMED	nifti, bvals, bvecs<br>nifti, bvals and bvecs in opposite phase encoding	FA<br>MD<br>RD<br>RSF
QMT	11 MT weightings + 0 MT weighting	MMPF<br>MTR
McDespot	ssfp_180<br>ssfp_0<br>spgr<br>spgr_IR	MWF<br>R1

**MMMP and MWF not available in one session*
