# Supplementary material for: MICRA: Microstructural image compilation with repeated acquisitions
Source: Neuroimage. 2021 Jan 15;225:117406. doi: 10.1016/j.neuroimage.2020.117406 (PMC7779421; doi:10.1016/j.neuroimage.2020.117406)
Supplement: Supplementary file 2 [file mmc2.pdf]

Sample size estimations calculations

Sample size estimations were computed using G*Power (Faul et al., 2009).

Percentage differences were first computed by calculating 1% and 5% of the mean each microstructural metric.

For the independent groups t-test:

The percentage change and standard deviations (assumed equal across two groups) were used as inputs in G*Power as shown below. Significance alpha was set to .05 and Power was set to .8.

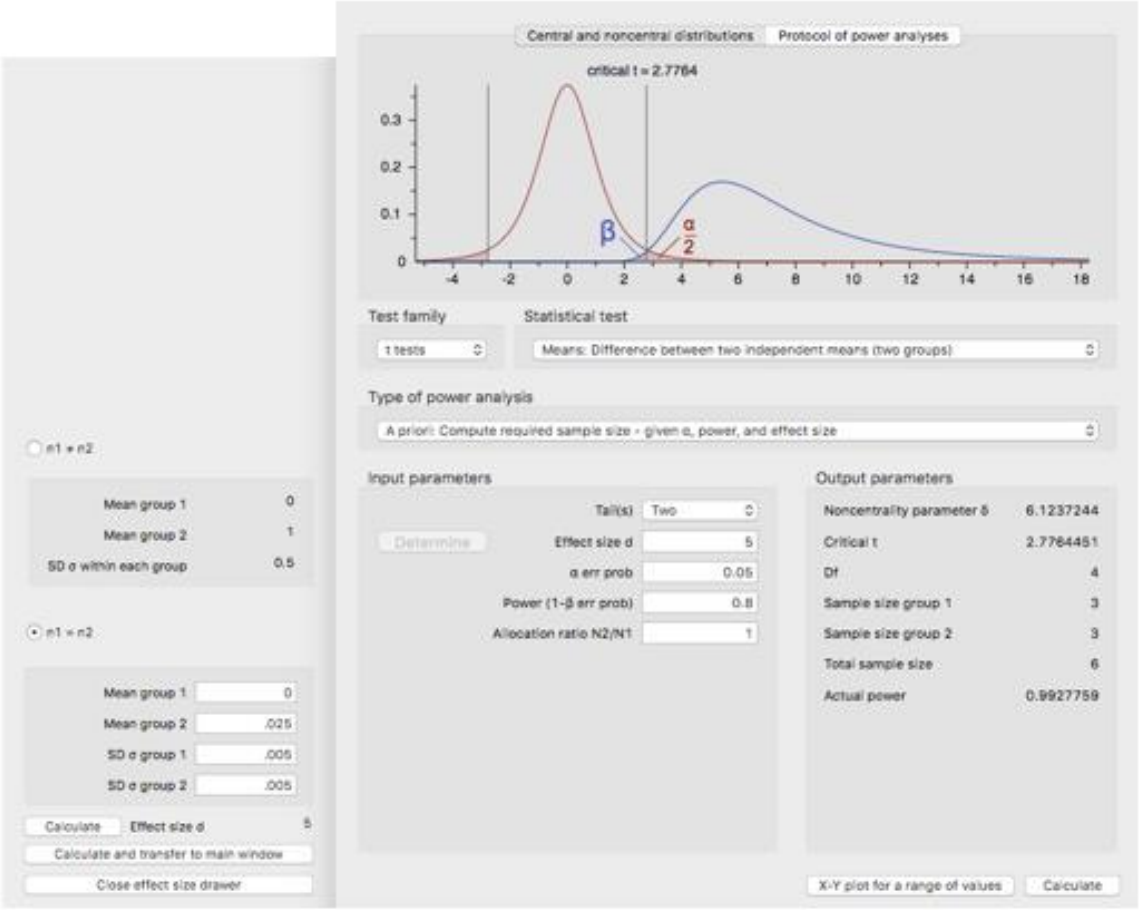

For the Group (2) x Time (2) between-within groups ANOVA:

Pearson correlation coefficients were used to account for the correlation among repeated measures for sample size estimation (see Table 2). Tests were computed using small (.1), medium (.25) and large (.4) effect sizes. Significance alpha was set to .05 and Power was set to .8.

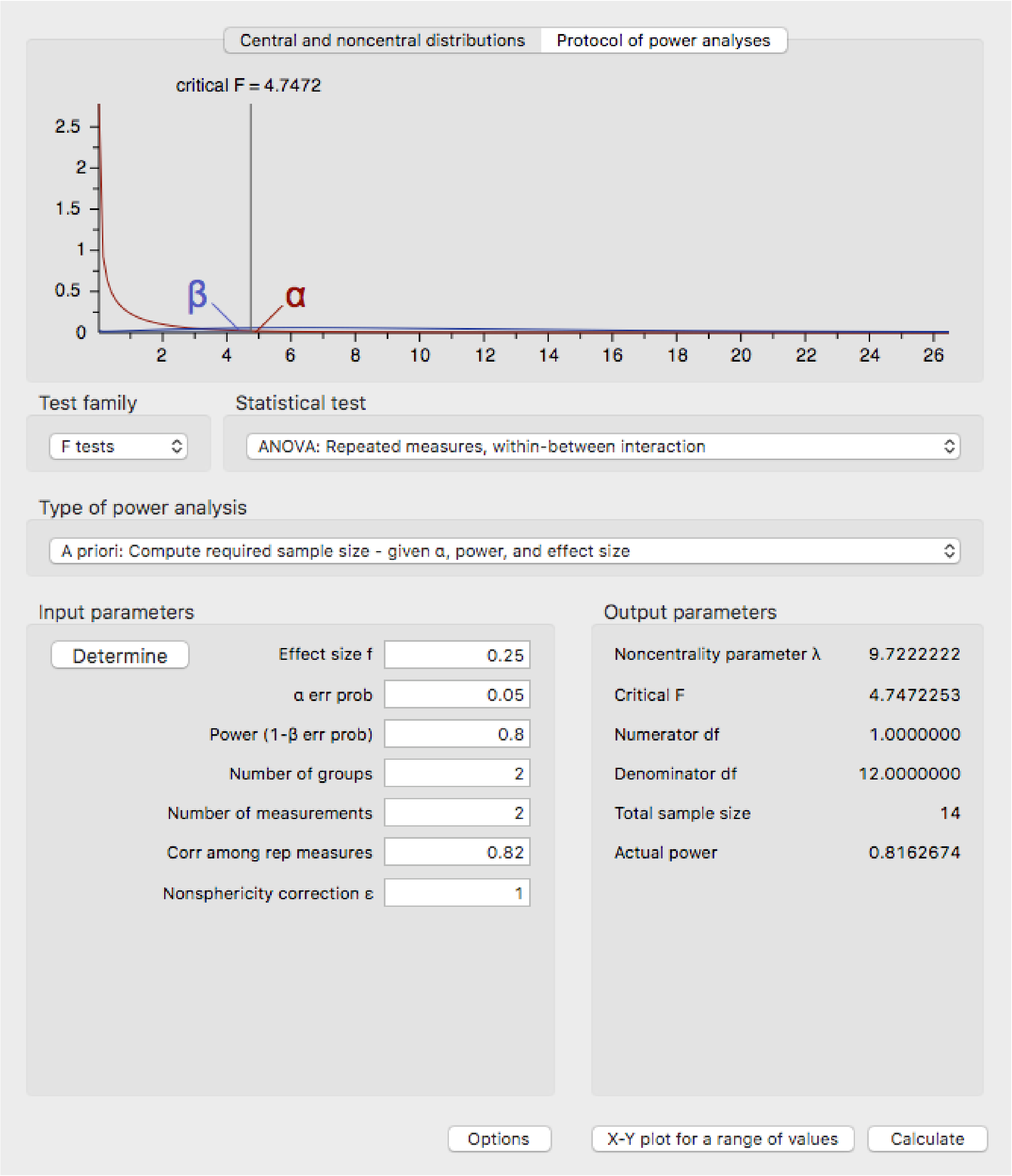

References:

Faul, F., Erdfelder, E., Buchner, A., & Lang, A.-G. (2009). Statistical power analyses using G*Power 3.1: Tests for correlation and regression analyses. Behavior Research Methods, 41, 1149-1160.
